# Tailoring the Grain Size of Bi-Layer Graphene by Pulsed Laser Deposition

**DOI:** 10.3390/nano8110885

**Published:** 2018-11-01

**Authors:** Jin Wang, Xuemin Wang, Jian Yu, Tingting Xiao, Liping Peng, Long Fan, Chuanbin Wang, Qiang Shen, Weidong Wu

**Affiliations:** 1 State Key Lab of Advanced Technology for Materials Synthesis and Processing, Wuhan University of Technology, Wuhan 430070, China; swustwj@163.com (J.W.); wangcb@whut.edu.cn (C.W.); 2 Science and Technology on Plasma Physics Laboratory, Research Center of Laser Fusion, China Academy of Engineering Physics, Mianyang 621900, China; wangxuemin75@sina.com (X.W.); yujianroy@163.com (J.Y.); tingtingxiao@yeah.net (T.X.); pengliping2005@126.com (L.P.); sfanlong@163.com (L.F.); 3 Collaborative Innovation Center of IFSA (CICIFSA), Shanghai Jiao Tong University, Shanghai 200240, China

**Keywords:** graphene, PLD, mobility

## Abstract

Improving the thermoelectric efficiency of a material requires a suitable ratio between electrical and thermal conductivity. Nanostructured graphene provides a possible route to improving thermoelectric efficiency. Bi-layer graphene was successfully prepared using pulsed laser deposition in this study. The size of graphene grains was controlled by adjusting the number of pulses. Raman spectra indicated that the graphene was bi-layer. Scanning electron microscopy (SEM) images clearly show that graphene changes from nanostructured to continuous films when more pulses are used during fabrication. Those results indicate that the size of the grains can be controlled between 39 and 182 nm. A detailed analysis of X-ray photoelectron spectra reveals that the sp^2^ hybrid state is the main chemical state in carbon. The mobility is significantly affected by the grain size in graphene, and there exists a relatively stable region between 500 and 800 pulses. The observed phenomena originate from competition between decreasing resistance and increasing carrier concentration. These studies should be valuable for regulating grains sizes for thermoelectric applications of graphene.

## 1. Introduction 

With increasingly serious environmental pollution and an energy crisis, it is very important to reduce environmental pollution and convert waste heat into electrical energy. For this reason, it is necessary to find efficient thermoelectric conversion materials. Excellent thermoelectric efficiency requires high electrical conductivity and low thermal conductivity. Nanostructured materials [1] limit the mean free path of electrons while restricting heat conduction. This shows that the electrical properties of nanomaterials are related to their special structures [2,3,4]. Nanostructured graphene has special electrical transport properties and is expected to have high thermoelectric efficiency [5,6,7,8]. Previous studies show that nanostructured graphene can provide significantly reduced thermal conductivity with little effect on electrical conductivity [9]. Thus, nanostructured graphene with controllable grain size can greatly improve the thermoelectric efficiency. Currently, the mainstream method for preparing graphene is chemical vapor deposition (CVD) [10,11,12,13]. Most researchers focus on the properties of single grain graphene, but the influence of crystal grain size on electrical conductivity of graphene is still unclear at the macroscopic scale [14,15,16,17]. The primary reasons for those observed phenomena originate from the fact that it is difficult to use CVD methods to adjust the size of graphene nanograins. Therefore, the preparation of graphene with controllable grain size is the key to expanding the applications of graphene, especially thermoelectric applications [18,19]. Because pulsed laser deposition (PLD) can be used to controllably generate highly energetic carbon species [18,19,20,21,22], it has natural advantages in controlling graphene crystal grains. This method is suitable for adjusting the size of graphene grains. Early experiments examined the effects of laser energy, substrate temperature, ablation time, and cooling rate [23,24,25,26,27]. However, research on the control of graphene crystal grains by PLD is still deficient.

Bi-layer graphene was prepared using PLD in this study. The effect of pulse numbers on the size of graphene grains was studied. In this case, the growth process of bi-layer graphene grains could be sufficiently controlled. 

## 2. Experimental 

Graphene grains were deposited on single crystal Cu (111) substrates by PLD. An excimer KrF laser was used for ablation. The specific experimental parameters are listed in Table 1. The number of pulses was set to 300, 500, 700, 800, and 900, and the corresponding samples are labeled in Table 2. Raman spectra from the graphene samples were gathered using a 514-nm laser in backscattering geometry at room temperature (Invia, Renishaw, London, UK). A field emission scanning electron microscopy (FE-SEM) (Quanta 250, FEI, Hillsboro, OR, USA) operated at 15 kV was used to examine the surface morphology of graphene and view the graphene grains. The working distance was 5 mm. The grain size distribution and average grain size of graphene were calculated using Nano Measurer software (Nano Measurer v1.2.5). We measured graphene grains with different sizes and in different regions in the SEM images in order to ensure accurate results. X-ray photoelectron spectra (XPS) spectra were gathered with an ESCALAB 250Xi XPS while the samples were excited with Al *K*α radiation. The electrical properties of graphene were determined from Hall measurements.

## 3. Results and Discussion

**Figure 1:**
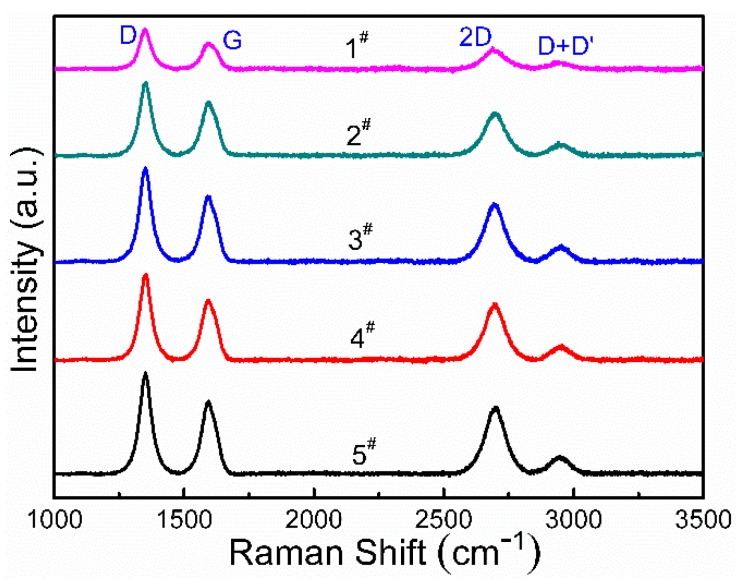
Raman spectra of graphene from samples of 1^#^–5^#^.

Figure 1 shows the Raman spectra from the graphene films deposited on the single crystal Cu (111) surface. Four peaks are present in the Raman spectra. The strong D peak at 1350 cm^−1^ was induced by disorder in the atomic arrangement, the edge effect of graphene, or ripples and charge puddles. This means a significant number of defects appeared in the graphene thin films. The G peak at approximately 1580 cm^−1^ originates from highly oriented graphite induced by the doubly degenerate zone center E_2g_ mode. The 2D peak at approximately 2700 cm^−1^ originates from the double resonance Raman excitation of two-photon near two mutually nonequivalent *K* points at the center of the first Brillouin zone. The intensities of *I*_G_, *I*_2D_, and their ratios are useful indicators of the quality and number of layers in the graphene samples. The specific peak information, *I*_D_/*I*_G_, and *I*_2D_/*I*_G_ ratios are shown in Table 3. As the number of pulses increases, the peak intensity of the D, G, and 2D peaks in the Raman spectra constantly increased. The ratio of *I*_2D_/*I*_G_ fell in between 0.79 and 0.94, implying the graphene layers have a bi-layer structure [28,29,30,31]. This means that the number of graphene layers remains constant as the number of pulses increases. One possible reason is that single crystal Cu (111) may play a role in limiting or preventing precipitation altogether at 1000 °C [32]. Another peak in Figure 1 at approximately 2960 cm^−1^ (called D + D’) is a dual-phonon process peak originating from one intravalley and one intervalley phonon scattering [33]. This peak is closely related to the defect state. The peak intensity increased significantly as the defect density increased. Interestingly, the D + D’ peak is only observed in graphene prepared by PLD, and the peak has not been observed in graphene films prepared with other methods. 

**Figure 2:**
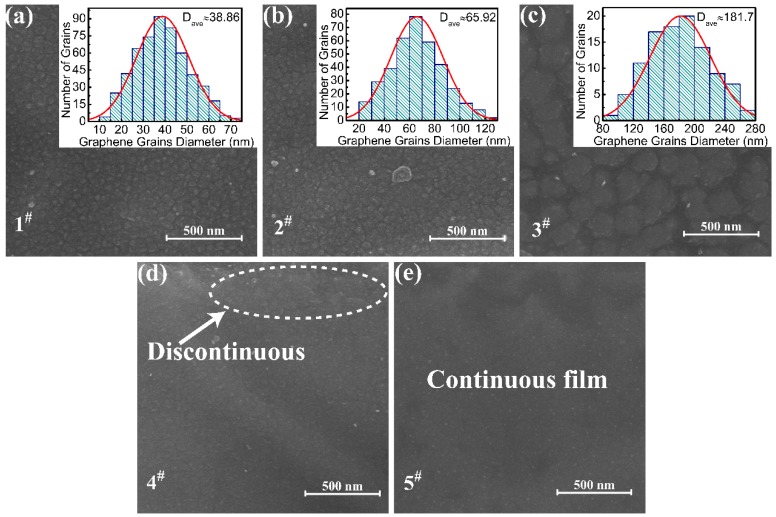
Scanning electron microscopy (SEM) image of (**a**) sample 1^#^, (**b**) sample 2^#^, (**c**) sample 3^#^, (**d**) sample 4^#^, and (**e**) sample 5^#^. The inset in (**a**–**c**) show the corresponding grain size distribution. The white dotted ellipse in (**d**) shows the discontinuous part.

**Figure 3:**
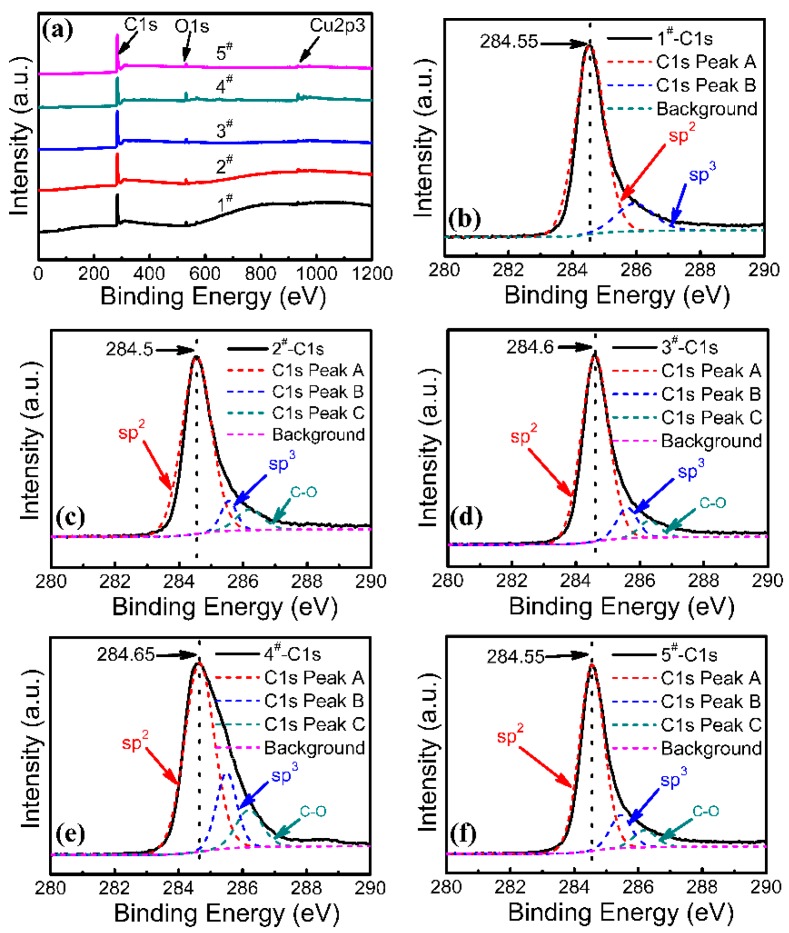
(**a**) X-ray photoelectron spectra (XPS) spectra from graphene grown using different number of pulses. (**b**–**f**) C 1s peaks in graphene from samples 1^#^–5^#^, respectively.

The morphology of graphene is clearly shown in the SEM image in Figure 2. Figure 2a–e show SEM images of graphene from samples of 1^#^–5^#^, respectively. Sample 1^#^ contains small and discontinuous graphene grains. The corresponding grain size distribution is shown in Figure 3a, where the average graphene grain size is 39 nm. The formation of graphene nanocrystals is caused by multiple nucleation sites on the surface of the Cu (111) substrate at a small number of pulses. The step on the Cu (111) surface results from high temperature. As shown in sample 2^#^, it was found that graphene nanograins are connected to each other to form graphene grains with an average size of 66 nm when the number of pulses increased to 500. Although the small grains are connected to each other to form larger graphene grains, it can be seen from the figure that there are still many discontinuities. Sample 3^#^ was grown using 700 pulses, resulting in large graphene grains with an average size of approximately 182 nm. In sample 4^#^, the number of pulses increased to 800. It can be seen that the graphene grains are almost all connected together to form a continuous film in Figure 3e, but there are still discontinuities shown in the white dotted ellipse. In sample 5^#^, full coverage is achieved, indicating that a completely continuous graphene film was formed. The size of bi-layer graphene grains was controlled by adjusting the number of pulses. Nanostructured graphene prepared by PLD growth gives hope that one would have a much better control of the thermal properties of supported bi-layer graphene since the grain size has an effect on *K* (*T*) of graphene. Recently, studies [34,35,36] show that acoustic flexure (ZA) modes are the dominant heat transport in graphene based on the dependence of *K* (*T*) ~*T*^1.4^ or ~*T*^1.5^. That means *K* can be adjusted within a certain range by controlling the graphene structure.

XPS measurements [37] can provide direct evidence of the chemical states in graphene. Figure 3 shows the XPS spectra from graphene grown using different numbers of pulses. Figure 3a shows XPS spectra from each sample, which indicate the existence of C, O, and Cu. The main features correspond to C 1s, O 1s, and Cu 2p3. The major species remaining were C=C (284.7 eV). The C 1s spectra from all samples are shown in Figure 3b–f, respectively. Peak A at 284.7 eV (C 1s) is attributed to sp^2^ carbon bonds, which agrees with the component of graphene [38]. It is well known that graphene formation occurs due to the surface graphitization of carbon films. Peak B at 285.50.1 eV corresponds to sp^3^ carbon atoms. Peak C exhibits much smaller intensity at about 286.3 eV and is attributed to some C–O contamination at the surface of the films due to exposure to air [39]. The XPS results show that the growth kinetic energy provided by the PLD system cannot induce a complete transformation of all sp^3^ bonds into sp^2^ bonds in graphene. These results indicate the presence of growth defects during graphene preparation using PLD.

**Figure 4:**
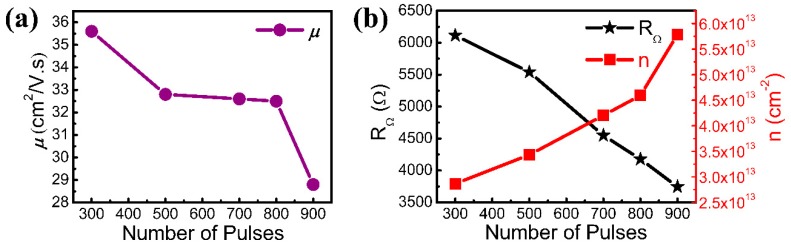
(**a**) Room-temperature mobility of graphene with different number of pulses; (**b**) *n* and *R*_Ω_ of graphene with the samples of 1^#^, 2^#^, 3^#^, 4^#^, and 5^#^, respectively.

Figure 4a shows the room-temperature mobility of graphene with different numbers of pulses measured by the Hall effect. This clearly implies that the measured graphene mobility is very low in the experiment. The low mobility of graphene occurs due to grain boundaries and defects. Meanwhile, the mobility of graphene is basically stable, especially when grown using between 500 and 800 pulses. The mobility increases as the number of pulses used during growth decreases. The mobility of graphene is determined using the formula μ=σ/ne, where *σ* is the electrical conductivity [40]. σ=1/ρ, where *ρ* is the resistivity. Therefore, the graphene mobility formula can be simplified as $\mu =1/R_{\Omega }\cdot n\cdot e$, where *R*_Ω_ is the sheet resistance and *n* is the carrier concentration. The relationship between *R*_Ω_ and *n* and the number of pulses used during growth was studied in order to better understand its effect on mobility. Figure 4b shows that the carrier concentration *n* is ~10^13^, which is one to two orders of magnitude higher than graphene with good mobility [41]. The measured resistance of the graphene samples is several kΩ. The two factors result in low mobility. With the increasing number of pulses, it can be seen from the previous Raman spectra results that the defect peak D gradually increased, indicating that the defect state density in graphene increased. As the density of the defect state increased, *n* also increased. However, *R_Ω_* decreases with increasing grain size. The mobility *μ* becomes relatively stable when the competition between *R*_Ω_ and *n* is balanced. This is the reason why the mobility of graphene is basically stable when grown using 500 to 800 pulses.

## 4. Conclusions

In conclusion, we prepared bi-layer graphene from a solid carbon source using PLD. The grain size of graphene can be controlled between 39 and 182 nm by varying the number of pulses from 300 to 900. Regarding the chemical structure, sp^3^ bonds exist in graphene, which lead to many defects during graphene growth. Electronic mobility can be affected by grain size and becomes relatively stable between 500 and 800 pulses. These results may occur due to competition between resistance and carrier concentration. These findings can be used to tune the grain size of graphene, and the results are beneficial for thermoelectric applications.

## Figures and Tables

**Table 1:** Experimental fabrication parameters.

Experiment Conditions	Experimental Parameters
Background vacuum	2.0 × 10^−6^ Pa
Working vacuum	4.5 × 10^−5^ Pa
Target	highly oriented pyrolytic graphite (HOPG) (purity > 99.99%)
Substrate	single crystal Cu (111)
Laser pulse frequency	1 Hz
Energy density	4 J/cm^2^
Distance between the target and the substrate	10 cm
Annealing condition	1000 °C

**Table 2:** Sample numbers and their corresponding pulse numbers.

Samples	1^#^	2^#^	3^#^	4^#^	5^#^
Number of pulses	300	500	700	800	900

**Table 3:** Raman intensity for *I*_D_, *I*_G_, *I*_2D_, and the ratio of *I*_D_/*I*_G_ and *I*_2D_/*I*_G_ from Figure 1.

Samples	D-Band Position	D-Band Intensity	G-Band Position	G-Band Intensity	2D-Band Position	2D-Band Intensity	*I*_D_/*I*_G_	*I*_2D_/*I*_G_
1^#^	1349 cm^−1^	3240	1598 cm^−1^	2103	2688 cm^−1^	1728	1.54	0.82
2^#^	1354 cm^−1^	5922	1595 cm^−1^	4333	2690 cm^−1^	3426	1.37	0.79
3^#^	1350 cm^−1^	7572	1592 cm^−1^	5262	2693 cm^−1^	4683	1.44	0.89
4^#^	1354 cm^−1^	6913	1595 cm^−1^	4834	2693 cm^−1^	4528	1.43	0.94
5^#^	1350 cm^−1^	8143	1595 cm^−1^	5849	2704 cm^−1^	5359	1.39	0.92
